# The impact of medical tourism on cervical cancer screening among immigrant women in the U.S.

**DOI:** 10.1186/s12905-021-01558-0

**Published:** 2021-12-15

**Authors:** Sou Hyun Jang, Hendrika Meischke, Linda K. Ko

**Affiliations:** 1 Department of Sociology, Sungkyunkwan University, 25-2 Sungkyunkwan-ro, Jongno-gu, Seoul, South Korea; 2 Department of Health Systems and Population Health, Hans Rosling Center for Population Health, University of Washington, 3980 15th Avenue NE, 4th Floor, UW Mailbox, Seattle, WA 351621 USA; 3 Division of Public Health Sciences, Fred Hutchinson Cancer Research Center, 1100 Fairview Ave N., Mail Stop M3-B232, Seattle, WA USA

**Keywords:** Cervical cancer screening, Pap smear, Immigrant women, Recent immigrants, Medical tourism, Self-sample HPV test

## Abstract

**Background:**

Research on the relationship between medical tourism—traveling abroad for healthcare and cervical cancer screening is lacking. This study examines (1) the association between medical tourism and cervical cancer screening among immigrant women and (2) whether the association varies across years in the U.S.

**Methods:**

We analyzed the New Immigrant Survey data of immigrant women aged 21–65 (n = 999). The outcome was having had a Pap smear since becoming a permanent resident, and the main predictor was medical tourism. Logistic regressions were conducted.

**Results:**

Immigrant women who engaged in medical tourism had higher cervical cancer screening rates compared to those who did not engage in medical tourism (84.09% vs. 71.68%). This relationship was statistically significant only among women who have recently immigrated, after controlling for covariates.

**Conclusions:**

Immigrant women who engaged in medical tourism had 2.18 higher odds of receiving a Pap smear than immigrant women who did not, after controlling for other covariates. Health educators should be aware of the practice of medical tourism and consider providing education on adherence to cancer screening guidelines and follow up abnormal results to ensure that immigrant women receive continuous cancer care.

## Background

Duration of stay in the United States (U.S.) is one of the main factors associated with immigrant women’s cancer screenings. Most studies on immigrant women show that those who have lived in the U.S. longer have higher cervical cancer screening rates, compared to those who have lived in the U.S. for a shorter duration [1–6]. For example, in contrast to 83% of immigrant women who have lived in the U.S. for ten years or longer, only 73% of those who have lived in the U.S. for less than ten years have received a Pap smear in the last two years [7]. Immigrant women who have lived in the U.S. for ten years or longer have approximately 1.4 higher odds of cervical cancer screening than those who have lived in the U.S. for less than ten years [8].

A new body of literature suggests that some immigrant groups engage in medical tourism (i.e., visiting a foreign country—usually their home country—for healthcare). Whereas medical tourism is not a new phenomenon, globalization has accelerated it worldwide [9]. Medical tourism can be divided into two types: general medical tourism and immigrant medical tourism [10]. General medical tourism refers to an individual’s trip to *any* country, other than their own, to receive medical care. For example, this type includes citizens from the United Kingdom or the U.S. seeking medical care in Thailand, India, or Singapore because of affordability of medical care, while also visiting the country and therefore, receiving “medical” services as well as “tourism” experience [11, 12]. By contrast, immigrant medical tourism refers to immigrants’ returning to their home country to receive medical care [10]. Recent immigrants report experiencing more barriers (e.g., health beliefs, language barriers, cultural values and attitudes, and having no health insurance) to accessing healthcare in the U.S. than their non-recent immigrant counterparts [7, 13–17]. Research shows that immigrants who have recently immigrated to the U.S. are likely to maintain closer transnational ties (e.g., contact with family members and friends) with their home country than their non-recent counterparts [18, 19]. When these immigrants encounter barriers to seeking healthcare in the U.S., such as language and cultural barriers, they are more likely to return to the familiar and cost-saving healthcare system in their home country [10, 20].

Earlier studies on immigrant medical tourism in the U.S. largely focused on Mexican and Korean immigrants [10, 21–29]. Researchers have found that more than 10% of Mexican immigrants in the U.S. have undertaken in medical tours to their home country [25, 27]. Whereas Mexican immigrants who live in border states are more likely to travel to their home country to seek medical care [28], medical tourism has also been documented among those living outside of border states [21]. Factors associated with participation in medical tourism among Mexican immigrants, include affordable medical care in the home country, lack of health insurance in the U.S., and familiarity with the home country’s healthcare system [21, 22, 27, 28]. Although these are also the reasons for Korean immigrants to engage in medical tourism [10, 24, 29], their transnational ties with their home country (e.g., contacting family members and friends in the home country) are also associated with medical tourism [10].

The use of medical tourism for cancer screening is increasing among immigrants [23, 26, 30]. For example, Korean immigrants engaged in medical tourism were approximately nine times more likely to be up-to-date with colorectal cancer screening [23] and approximately five times more likely to be up-to-date with breast and cervical cancer screenings [30], compared to those who did not engage in medical tourism. However, there is insufficient research that captures the engagement in medical tourism across different racial/ethnic groups or with years lived in the U.S. This study aims to examine (1) whether medical tourism is associated with cervical cancer screening among immigrant women and (2) whether the association between medical tourism and cervical cancer screening varies by years lived in the U.S.

## Methods

### Data

This study used publicly available secondary data, the New Immigrant Survey (NIS), a multi-cohort longitudinal dataset of immigrants aged over 18 years, who have been permanent residents since 2003–2004. Based on the guidance of the University of Washington Institutional Review Board (IRB), this study was exempted from review. The NIS was conducted in two rounds: 2003–2004 (round 1) and 2007–2009 (round 2). In both rounds, respondents were asked various questions, including their socio-demographic characteristics, health status, and healthcare utilization. Our study merged the data from both the rounds. We used socio-demographic characteristics from the first round because most respondents skipped these questions in the second round, as their characteristics remained unchanged. English proficiency and health insurance were also taken from the first round. The outcome and predictor variables were derived from the second round. Since the second round was conducted during 2007–2009, there was sufficient time for respondents to receive cancer screening in the U.S. after becoming a permanent resident or to travel abroad for medical care. We used the United States Preventive Services Task Force (USPSTF) recommendation for a Pap smear for cervical cancer screening every three years for women aged 21–65 years [31]; therefore, this study included immigrant women within that age bracket (n = 999).

### Measures

The outcome variable in this study was having undergone cervical cancer screening since becoming a permanent resident. To measure this, we used the following question: “Since Round 1, have you had a Pap smear?” Responses included yes or no.

The main predictor was medical tourism. This variable was captured in the second round of the NIS with the question: “Did you visit a doctor in the U.S. or a foreign country?” Response categories included (1) having visited a doctor in the U.S., (2) having visited a doctor in a foreign country, or (3) having visited a doctor in the U.S. and a foreign country. If a respondent reported visiting a doctor in a foreign country or both in the U.S. and a foreign country, they were classified as having participated in medical tourism.

Other predictors included socio-demographic characteristics (e.g., race/ethnicity, age, and education), having health insurance, self-rated health status, diagnoses of chronic illness, and English proficiency. We also included respondents’ state of residence, and considered six states that have the highest immigrant population: California, New York, New Jersey, Texas, Florida, and Illinois [32].

A cutoff of ten years was used to categorize immigration status; this cutoff was in line with previous studies that reported that the first ten years of immigration are critical for cultural assimilation into the U.S. society and for acquiring English language proficiency [33, 34]. We subtracted the year of the survey from the beginning of the respondent’s residency, and immigrants were categorized into “recent” (having lived in the U.S. for less than ten years) and “non-recent” (having lived in the U.S. for ten years or more).

### Statistical analysis

All analyses were conducted using Stata 15.0. For multivariate analyses, logistic regression models were built to examine the association between medical tourism and cervical cancer screening, controlling for other covariates that have been found to be associated with cancer screening. Each logistic regression model was applied to the two groups (recent vs. non-recent immigrants). Statistical tests were considered significant at *p* = 0.05.

## Results

Table 1 shows the characteristics of the survey participants by years lived in the U.S. The mean age of the sample (aged 21 to 65) was 41.55 years (SD = 10.17). By age, approximately 13.01% were aged 21–29, 33.43% aged 30–39, 30.83% aged 40–49, 17.82% aged 50–59, and 4.9% aged 60–65. By race and ethnicity, the largest group was Hispanic (36.94%), followed by non-Hispanic Asian and Pacific Islanders (31.83%), non-Hispanic whites (21.02%), and non-Hispanic blacks (10.21%). Considering the top five states of residence, most respondents lived in California (27.83%), followed by Texas (8.81%), Florida (8.70%), Illinois (7.19%), and New York (6.28%); among “other” states, New Jersey (9.31%) accounted for the most respondents. The majority were married (71.87%), had no college education (70.57%), and were employed (62.11%). Approximately half (48.65%) were insured and most (76.38%) reported being in good, very good, or excellent health. As many as 13.11% had one or more diagnoses of chronic illnesses, and 8.81% of respondents engaged in medical tourism after becoming permanent residents.

**Table 1:** Characteristics of survey participants by years lived in the U.S. (N, %)

	Total (n = 999)	Non-recent (n = 346)	Recent (n = 653)	*P* value
Age; Mean (SD)	41.55 (10.17)	41.85 (9.00)	41.39 (10.75)	0.032
Age groups				0.000
21–29	130 (13.01)	30 (8.67)	100 (15.31)	
30–39	334 (33.43)	115 (33.24)	219 (33.54)	
40–49	308 (30.83)	133 (38.44)	175 (26.80)	
50–59	178 (17.82)	56 (16.18)	122 (18.68)	
60–65	49 (4.90)	12 (3.47)	37 (5.67)	
Race/ethnicity				0.000
White	210 (21.02)	46 (13.29)	164 (25.11)	
Black	102 (10.21)	22 (6.36)	80 (12.25)	
Hispanic	369 (36.94)	214 (61.85)	155 (23.74)	
Asian and Pacific Islanders	318 (31.83)	64 (18.50)	254 (38.90)	
State of residence (n = 988)				0.000
California	275 (27.83)	123 (35.86)	152 (23.57)	
Florida	86 (8.70)	30 (8.75)	56 (8.68)	
Illinois	71 (7.19)	17 (4.96)	54 (8.37)	
New York	62 (6.28)	18 (5.25)	44 (6.82)	
New Jersey	92 (9.31)	30 (8.75)	62 (9.61)	
Texas	87 (8.81)	38 (11.80)	49 (7.60)	
Other	315 (31.88)	87 (25.36)	228 (33.35)	
Marital status				0.030
Unmarried	281 (28.13)	112 (32.37)	169 (25.88)	
Married	718 (71.87)	234 (67.63)	484 (74.12)	
Education status				0.000
Lower than BA	705 (70.57)	271 (78.32)	434 (66.46)	
BA or higher degree	294 (29.43)	75 (21.68)	219 (33.54)	
Employment status				0.646
Unemployed	377 (37.89)	127 (36.92)	250 (38.40)	
Employed	618 (62.11)	217 (63.08)	401 (61.60)	
English proficiency				0.005
Not well/not at all	645 (64.56)	203 (58.67)	442 (67.69)	
Well/very well	354 (35.44)	143 (41.33)	211 (32.31)	
Having health insurance				0.101
No	513 (51.35)	190 (54.91)	323 (49.46)	
Yes	486 (48.65)	156 (45.09)	330 (50.54)	
Current health status				0.011
Fair/poor	236 (23.62)	98 (28.32)	138 (21.13)	
Good/very good/excellent	763 (76.38)	248 (71.68)	515 (78.87)	
Number of diagnoses				0.089
0	868 (86.89)	292 (84.39)	576 (88.21)	
1 or more	131 (13.11)	54 (15.61)	77 (11.79)	
Engagement in medical tourism				0.293
No	911 (91.19)	320 (92.49)	591 (90.51)	
Yes	88 (8.81)	26 (7.51)	62 (9.49)	

We observed clear differences between non-recent and recent immigrants by age, race/ethnicity, state of residence, marital status, education status, English proficiency, and health status. Most recent immigrant women were either the youngest (21–29 years old) or the oldest (50–59 and 60–65 years old) of the age groups, whereas most non-recent immigrant women were in the 30–39 and 40–49 age groups. Compared to their non-recent peers, recent immigrants were less likely to be Hispanic, more likely to live in a state on the East coast (e.g., Illinois, New York, and New Jersey), to be married, more educated, have lower English proficiency, and have an excellent health status. Engagement in medical tourism was not statistically significant between non-recent (7.51%) and recent immigrants (9.49%).

In the bivariate analyses, age, race/ethnicity, state of residence, English proficiency, years lived in the U.S., having health insurance, having one or more diagnoses of a chronic illness, and engaging in medical tourism were significantly associated with immigrant women’s cervical cancer screening rates (Table 2). Immigrants aged 30–49 were more likely to have had cervical cancer screening than immigrants aged 21–29 or 50–65. Hispanic immigrants, those who resided in California and Florida, and those who spoke English well or very well, were more likely to have had cervical cancer screening than their counterparts. Furthermore, non-recent immigrants had higher cervical cancer screening rates than recent immigrants (85.26% vs. 66.16%; *p* = 0.000). Additionally, immigrants who have undergone one or more diagnoses of chronic illnesses had higher cervical cancer screening rates than those without any diagnoses, as did those who engaged in medical tourism compared to those who did not (84.09% vs. 71.68%; *p* = 0.012).

**Table 2:** Bivariate relationship between the predictors and cervical cancer screening (N, %)

	All (n = 999)	Not Screened (n = 272)	Screened (n = 727)	*P* value
Age group				0.004
21–29	130 (13.01)	40 (14.71)	90 (12.38)	
30–39	334 (33.43)	77 (28.31)	257 (35.35)	
40–49	308 (30.83)	73 (26.84)	235 (32.32)	
50–59	178 (17.82)	66 (24.26)	112 (15.41)	
60–65	49 (4.90)	16 (5.88)	33 (4.54)	
Race/ethnicity				0.000
White	210 (21.02)	62 (29.52)	148 (70.48)	
Black	102 (10.21)	43 (42.16)	59 (57.84)	
Hispanic	369 (36.94)	46 (12.47)	323 (87.53)	
Asian and Pacific Islanders	318 (31.83)	121 (38.05)	197 (61.95)	
State of residence (n = 988)				0.002
California	275 (27.83)	59 (22.01)	216 (30.00)	
Florida	86 (8.70)	12 (4.48)	74 (10.28)	
Illinois	71 (7.19)	26 (9.70)	45 (6.25)	
New York	62 (6.28)	18 (6.72)	44 (6.11)	
New Jersey	92 (9.31)	23 (8.58)	69 (9.58)	
Texas	87 (8.81)	26 (9.70)	61 (8.47)	
Other	315 (31.88)	104 (38.81)	211 (29.31)	
Marital status				0.134
Unmarried	281 (28.13)	86 (30.60)	195 (69.40)	
Married	718 (71.87)	186 (25.91)	532 (74.09)	
Education status				0.346
Lower than BA	705 (70.57)	198 (28.09)	507 (71.91)	
BA or higher degree	294 (29.43)	74 (25.17)	220 (74.83)	
Employment status				
Unemployed	377 (37.89)	106 (28.12)	271 (71.88)	0.666
Employed	618 (62.11)	166 (26.86)	452 (73.14)	
English proficiency				
Not well/not at all	645 (64.56)	190 (29.46)	455 (70.54)	0.033
Well/very well	354 (35.44)	82 (23.16)	272 (76.84)	
Years lived in the U.S				0.000
Less than 10 years	653 (65.37)	221 (33.84)	432 (66.16)	
10 years or longer	346 (34.63)	51 (14.74)	295 (85.26)	
Having health insurance				0.000
No	513 (51.35)	169 (32.94)	344 (67.06)	
Yes	486 (48.65)	103 (21.19)	383 (78.81)	
Current health status				0.427
Fair/poor	236 (23.62)	69 (25.37)	167 (22.97)	
Good/very good/excellent	763 (76.38)	203 (74.63)	560 (77.03)	
Number of diagnoses				0.025
0	868 (86.89)	247 (28.46)	621 (72.54)	
1 or more	131 (13.11)	25 (19.08)	106 (80.92)	
Engagement in medical tourism				0.012
No	911 (91.19)	258 (28.32)	653 (71.68)	
Yes	88 (8.81)	14 (15.91)	74 (84.09)	

SD, standard deviation

In the multivariate model, the results showed that, after adjusting for other covariates, Hispanic ethnicity, state of residence, years lived in the U.S., having health insurance, diagnoses of chronic illness, and engagement in medical tourism were all significantly associated with cervical cancer screening rates in the entire sample (Table 3). Hispanic immigrant women showed higher odds of having undergone cervical cancer screening than non-Hispanic white women. Those who resided in Texas and other states were less likely to have had a screening than those living in California. Furthermore, immigrant women who have lived in the U.S. for ten or more years, those who have health insurance, and those with one or more diagnoses of chronic illness were more likely to receive cervical cancer screening compared to their counterparts. Additionally, immigrant women who engaged in medical tourism were more likely to have had cervical cancer screening than those who did not (OR = 2.18, 95% CI = 1.11–4.26; *p* < 0.05).

**Table 3:** Multivariate Relationship between the Predictors and Cervical Cancer Screening

	All (n = 988)	Non-recent (n = 343)	Recent (n = 645)
Age groups			
21–29	1 (ref)	1 (ref)	1 (ref)
30–39	1.21 (0.73–1.98)	2.67 (0.81–8.73)	1.03 (0.59–1.78)
40–49	1.03 (0.61–1.72)	1.51 (0.47–4.88)	0.95 (0.53–1.69)
50–59	0.57 (0.32–1.01)	0.71 (0.20–2.53)	0.54 (0.28–1.03)
60–65	0.72 (0.31–1.64)	0.60 (0.09–3.71)	0.74 (0.29–1.90)
Race/ethnicity			
White	1 (ref)	1 (ref)	1 (ref)
Black	0.67 (0.38–1.18)	0.66 (0.17–2.54)	0.71 (0.37–1.34)
Hispanic	3.38 (2.03–5.64)***	3.25 (1.07–9.90)*	3.96 (2.14–7.35)***
Asian and Pacific Islanders	0.68 (0.44–1.04)	1.05 (0.31–3.47)	0.61(0.38–0.98)*
State of residence			
California	1 (ref)	1 (ref)	1 (ref)
Florida	1.33 (0.63–2.82)	4.40 (0.49–39.24)	0.95 (0.40–2.23)
Illinois	0.62(0.32–1.18)	0.32 (0.08–1.28)	0.69 (0.33–1.43)
New York	0.72 (0.36–1.45)	1.92 (0.33–11.01)	0.55 (0.25–1.23)
New Jersey	0.67 (0.36–1.26)	0.34 (0.10–1.13)	0.81 (0.39–1.68)
Texas	0.43(0.23–0.709)**	0.33 (0.11–0.98)*	0.42 (0.19–0.92)*
Other	0.63 (0.41–0.98)*	0.84 (0.33–2.15)	0.57 (0.34–0.96)*
Education status			
Less than BA	1 (ref)	1 (ref)	1 (ref)
BA degree or higher	1.32 (0.91–1.92)	1.03 (0.40–2.65)	1.35 (0.88–2.06)
English proficiency			
Not well	1 (ref)	1 (ref)	1 (ref)
Very well	1.54 (1.06–2.25)	0.83 (0.36–1.88)	1.78 (1.15–2.76)**
Years lived in the U.S			
Less than 10 years	0.53 (0.35–0.79)*	–	–
10 years or longer	1 (ref)	–	–
Having health insurance			
No	1 (ref)	1 (ref)	1 (ref)
Yes	2.16 (1.55–2.01)***	3.44 (1.53–7.70)**	1.95 (1.34–2.84)***
Number of diagnoses			
0	1 (ref)	1 (ref)	1 (ref)
1 or more	2.08 (1.22–3.54)**	1.06 (0.39–2.88)	2.62(1.39–4.94)**
Engagement in medical tourism			
No	1 (ref)	1 (ref)	1 (ref)
Yes	2.18 (1.11–4.26)*	2.78 (0.57–13.45)	2.19 (1.03–4.56)*
Intercept	2.34 (1.10–4.96)*	1.82 (0.35–9.45)	1.40 (0.68–2.88)
Pseudo R^2^	0.1531	0.1499	0.1284

OR, odds ratio; 95% CI, confidence interval

***p < .001; **p < .01; *p < .05

For both non-recent and recent immigrants, being Hispanic and having health insurance were positively associated with cervical cancer screening, whereas living in Texas was negatively associated. Among recent immigrants, Asian or Pacific Islanders who live in other states were less likely to receive cervical cancer screening, whereas recent immigrants with higher levels of English proficiency, one or more diagnoses, and engagement in medical tourism were more likely (OR = 2.19, 95% CI = 1.03–4.56; *p* < 0.05).

## Discussion

This study found that immigrant women who engaged in medical tourism had 2.18 higher odds of receiving a Pap smear than immigrant women who did not, after controlling for other covariates. While there was no significant difference in engagement in medical tourism between the non-recent and recent groups, a report of being recent immigrants was significantly associated with cervical cancer screening.

Recent immigrants may encounter more language and cultural barriers to navigate the U.S. healthcare system and may prefer procedures/screenings (including cervical cancer screenings) in other countries. Moreover, the impact of having health insurance on cervical cancer screening was much lower for recent immigrants than non-recent immigrants (OR = 1.95 vs. 3.44, respectively). One possible reason is that recent immigrants, who still have close ties with their home country, face barriers associated with being uninsured in the U.S., and thus return to their home country for cancer screening. Whereas medical tourism could help recent immigrants receive medical care in their home country when encountering barriers in the U.S., these individuals could benefit from health education on how to plan medical tourism for cervical cancer screening and be prepared for follow-up treatments, if the results are positive.

Studies have found that reports of chronic illnesses, such as hypertension, diabetes, and obesity, have a negative relationship with being up-to-date with cervical cancer screening among women in general [35–37], including immigrant women [38]. In contrast to earlier studies, our study found a positive relationship between having one or more chronic illnesses and cervical cancer screening. One possible explanation is that immigrant women with chronic illnesses may have a higher tendency to be more interested in their health or to visit the doctor more often as “the principal caregivers themselves” [39], which may positively influence their preventive behavior (cervical cancer screening). Future studies should further investigate why immigrant women with chronic illness report a higher rate of receiving a Pap smear and whether the type of chronic illness matters [36].

Previous studies found limited English proficiency to be one of the biggest obstacles encountered by immigrant women in receiving cervical cancer screening [14, 40, 41]. However, our findings indicate a significant association between English proficiency and cervical cancer screening only among recent immigrants. Studies have shown that immigrants’ English proficiency gradually increases, particularly over the first ten years [33, 34]; thus, immigrants who reported longer residence in the U.S. could have become more assimilated to the country, thereby lowering the language barriers involved in receiving cervical cancer screenings.

Whereas medical tourism could be beneficial in the short term, immigrant women may need to be educated on how to navigate the healthcare system in the U.S. and enroll in private or public insurance systems. Linking immigrants to community health centers could also be an option for those who cannot participate in the insurance system or those who are underinsured. Cervical cancer screening strategies, such as HPV self-sampling, which is more affordable and transferrable within the U.S. than medical tourism, may be more appealing to immigrant women, as it minimizes the interaction with the healthcare system [42, 43]. Similar to other cancer screening procedures, the introduction of HPV self-sampling may necessitate health education and discussion about a follow-up of abnormal results and subsequent cancer treatment in the U.S. [42].

This study has several limitations that could be addressed by future studies. First, it was not feasible to determine the foreign country in which the respondents had consulted a doctor. However, the answers still enabled us to determine whether they received care in the U.S. or a foreign country. Second, while it would be interesting to compare the cost of cancer screening and the national healthcare system between the U.S. and immigrants’ home countries, we did not include the respondents’ home countries in the analysis because of the small number of home countries in our sample. Third, the NIS includes only legal immigrants, who are permanent residents. Undocumented immigrants may face more barriers to receiving care (e.g., ineligibility to apply for federal health programs), and immigration-related challenges (e.g., fear of deportation) when receiving cancer screenings at a clinic or hospital. Future studies should investigate the factors associated with cancer screening among immigrants based on legal residency status. Lastly, there may have been changes in immigration and healthcare policies (e.g., Affordable Care Act) since the second round of NIS data. Thus, future rounds of data should help assess the impact of these policies on medical tourism and cervical cancer screening.

Nonetheless, this study has several strengths, such as a relatively large sample size, inclusion of all races and ethnicities, and the use of years lived in the U.S. to categorize immigrant women into recent and non-recent immigration groups. The latter enabled us to examine the association between medical tourism and cancer screening rates among immigrants after becoming permanent residents in the U.S.

This study is the first to examine medical tourism, years in the U.S., and cervical cancer screening across multiple racial/ethnic immigrant groups. Women with a recent immigrant status may be traveling abroad for cervical cancer screening. As medical tourism requires planning and could affect adherence to cancer screening guidelines and follow-up of abnormal results [10, 23, 30], more health education may be necessary to ensure that immigrant women receive continuous cancer care.

## Data Availability

The data is available at https://nis.princeton.edu/.
